# Report of a Case of Large Ovarian Tumor—Operation and Recovery

**Published:** 1895-08

**Authors:** Herman E. Hayd

**Affiliations:** Buffalo, N. Y., Attending Gynecologist to Erie County Hospital.


### REPORT OF A CASE OF LARGE OVARIAN TUMOR—OPERATION AND RECOVERY.

BY HERMAN E. HAYD, M. D., M. R. C. S., Eng., Buffalo, N. Y.,
Attending Gynecologist to Erie County Hospital.

VERY large ovarian tumors are comparatively rare, owing to the wonderful advance of modern surgery. Clearer methods of diagnosis, better equipment of the general practitioner and a more thorough appreciation of the pathology of pelvic disease has led to the performance of early operations for abdominal and pelvic troubles.

In China and oriental countries where medicine and surgery is still practised by the favored few, who become great and learned by reason of the ignorance and superstition of their people instead of by the possession of scientific knowledge, very large tumors and all kinds of monstrous pathological conditions are every day met with. Perhaps one of the most interesting cases of advanced ovarian disease occurred in China, and is reported in the April (1895) number of the *American Journal of Obstetrics*, where the woman weighed previous to the operation 246 pounds, and three weeks after operation seventy-seven pounds. She presented herself at the hospital door pushing a wheelbarrow in which she carried her own tumor.

Other cases are reported in our own country where the growth had reached enormous proportions, but perhaps none so large as this one alluded to.

The tumor in my case weighed between eighty and ninety pounds ; its fluid contents, thirty-four quarts, equal to sixty-eight pounds, and the sac and solid portions of the tumor eighteen pounds. The history of the case is, briefly, as follows :

Mrs. R., aged 55 ; married ; no children. First unwell when twenty ; no pain and regular. Five years ago she noticed a swelling in the right iliac region which was painless, and it progressively increased in size until she sought relief two years ago. A diagnosis of ovarian tumor was made and it was tapped and a large pailful of a limpid fluid was removed. It was tapped again in five months and about the same period for a dozen times or more, when it became necessary to tap it every two months ; but laterally it was done every three weeks and the amount of fluid which was removed each time more than filled two very large patten pails. In all she was tapped twenty-four times, and once I tapped her a few days before the operation so as to lessen the shock consequent upon the sudden removal of very large tumors. The fluid was of a greenish muddy color, thick and glutinous and run with difficulty through a good-sized trocar ; in fact, so slowly that after having taken away about fourteen quarts the instrument was withdrawn.

While exercising antiseptic precautions in the way of washing the skin and the like, the patient began to laugh and said, "That is not necessary. Push in the instrument, leave me a piece of sticking plaster and go away and I will pull it out when the tumor is empty."

Upon the right side, well up under the borders of the ribs, a large, hard mass could be made out, which, upon opening the belly, proved to be the solid part of the tumor. The belly was enormously distended and the skin and thinned abdominal parietes seemed to be quite adherent to the fluctuating mass, especially anteriorly, where numerous puncture marks were visible. Pain and tenderness were present in different areas. The woman's physical condition was fair, although she looked very much older than her years. The legs were somewhat swollen, but this swelling disappeared after remaining in bed a few days. Heart and lungs were normal and the kidneys showed no evidence of disease. For some days previous to operation hypodermic injections of strychnia, one-sixtieth grain, were given every fourth hour and the patient was put to bed and nourished freely with light but nutritious food.

On the morning of March 15, 1895, the operation was performed. A long incision was made and the cyst wall cut into and its contents let out. The adhesions anteriorly were so dense that it was a very difficult matter to decide which was abdominal wall and which was cyst. After considerable manipulation the peritoneal cavity was found and the adhesions, although free, were easily separated and the tumor delivered. Its pedicle was broad and it was tied off in sections with silk. The left ovary, which was the size of a very large orange and cystic, was also removed. The incision was then extended above to near the ensiform cartilage and below to the pubes, warm flat sponges were placed over the bowels; then about four inches of the redundant abdominal walls on each side of the incision were cut off and the wound brought together with silkworm-gut, after having first flushed the peritoneal cavity and placed a drainage-tube in position. There was some shock, but with the assistance of hypodermic injections of whisky and strychnia, together with the inhalation of oxygen gas, reaction came quickly. No vomiting or nausea of any moment followed the operation, and the tube, which discharged freely for the first twelve hours, was removed on the following morning. The temperature never rose above 99° and the whole course of the case was uninterrupted. The bowels moved on the third day after an injection of soap and water. The stitches were removed on the tenth day, on the twenty-third day the patient was permitted to sit up with a good fitting abdominal support and on the twenty-eighth left the hospital for her home in North Tonawanda.

The tumor was a large oöphoritic cyst, which contained a number of smaller cysts, and upon examination these smaller cysts simply contained a viscid glutinous fluid.

78 Niagara Street.

